# Deaths under Anæsthetics

**Published:** 1897-08-14

**Authors:** 


### Medical Progress and Hospital Clinics.

[*The Editor will be glad to receive offers of co-operation and contributions from members of the profession. All letters should be addressed to* The Editor, at the Office, 28 & 29, Southampton Street, Strand, London, W.C.]

### DEATHS UNDER ANÆSTHETICS.

Our last note on this unhappy topic was made on June 12th, when we recorded the dates and places of twelve deaths from chloroform which had occurred in England between April 19th and June 2nd. We now continue the list so far as the cases have come to our knowledge.

The *Sussex Daily News* for June 7th reports an inquest held by the Brighton Coroner, at the Sussex County Hospital, on a labourer who died under the influence of ether. An operation was about to be performed for " cancer of the bowels," presumably obstruction, as it was stated that at the post-mortem examination both heart and lungs were found to be compressed by the distended bowels.

The *Western Morning News*, June 16th, reports an inquest held at the Devon and Exeter Hospital on a young man, aged 18, who died whilst chloroform was being administered. It appears that after giving the chloroform for five minutes, all the staff being present, the administrator noticed that the pulse ceased to beat. The patient went on breathing for a little time longer, and means were taken to restore the heart's action, but without avail. He had not had enough chloroform to render him unconscious. Artificial respiration was kept up for three-quarters of an hour. At the post-mortem examination nothing was found the matter with any of the organs to account for the death.

The *Islington Gazette*, June 17th, reports an inquest on a child, aged 20 months, who died at the Great Northern Central Hospital while under chloroform. It seems that the child at first took it well, and after some four minutes the operation was commenced. Directly after this the breath stopped, and the heart failed. The operation was discontinued, and every effort was made to restore the child, but without avail. The patient had suffered from rickets, and on post-mortem examination it was found also to have had an affection of the heart.

The *Western Mercury*, June 18th, reports an inquest on a man, aged 28, who died at the South Devon and East Cornwall Hospital while under an anæsthetic, the nature of which is not mentioned, administered for the extraction of two teeth. He had previously had some tuberculous glands removed, and on post-mortem examination widespread tuberculosis was found to be present.

The *Daily News*, June 26th, reports an inquest held by the Blackburn Coroner on a youth, aged 17, who died while being put under chloroform for the removal of a growth in the neck. The operation had not been commenced when death took place.

The *South Wales Daily News*, July 13th, reports an inquest on a child, aged 9 years, who died under chloroform. The operation was for a severe injury to the hand, part of which had been blown away by a pistol. The operation was over, and as the stitches were being pu in the boy began to recover and to struggle. The puls then became weak, and breathing stopped. The death was attributed by witnesses to shock and loss of blood caused by the gunshot injury.

The *Kentish Express*, July 17th, reports an inquest on a man, aged 63, who died under chloroform at the Cottage Hospital, Ashford. The patient seems to have got under the influence of the anæsthetic, but the operation had not been commenced, when suddenly the heart stopped.

The *Birmingham Gazette*, July 26th, reports an inquest on a man, aged 29, who died under a mixture of alcohol, chloroform, and ether, which was given for tooth extraction. Nitrous oxide gas had been administered a few days before, but had not proved satisfactory. Before the extraction was commenced the pulse became feeble, and gradually ceased to beat. Efforts were made for an hour to restore the patient, but without avail.

The *Brixtonian*, July 24th, reports an inquest on a woman, aged 36, who died at the Clapham Maternity Home. She had been brought to the home for her confinement, the case being a difficult one. Chloroform was administered at 9.45, and was continued until 10.20, when the heart and pulse were good. Five minutes later, however, the heart failed and the patient died. One of the medical witnesses seems to have attributed the death to the chloroform, while the pathologist to the home attributed it to shock following the operation.

The *Birmingham Daily Post*, July 31st, reports an inquest on a man, aged 52, who died while under the influence of ether at the Wolverhampton Hospital. The patient was suffering from obstruction of the bowels; an operation was necessary, and it was performed under the influence of ether. Some minutes after the operation had been performed vomiting occurred; the vomited matter entered the glottis and suffocation resulted. A post-mortem examination showed that this was the cause of death.

This list includes some very interesting cases. Death from chloroform during labour is very rare, and the case here recorded, if the chloroform had anything to do with the death, is an exceptional one. In regard to the two deaths from ether, it is interesting to observe that they both occurred in abdominal cases. Again we have to draw attention to the comparative frequency with which death from chloroform occurs in the early stage, even before the commencement of the operation in some cases.

